# Scientific Items

**Published:** 1878-03-20

**Authors:** 


### *SCIENTIFIC ITEMS.*

### SUN-SPOTS AND TERRESTRIAL MAGNETISM.

1. The first coincidence observed was in the field of terrestrial magnetism. "A freely-suspended magnet, although it points in one direction, is nevertheless, within small limits, always in motion. Certain of these motions depend, as is well known, upon the hour of the day; but the magnet is also liable to irregular, abrupt fluctuations, which cannot be connected with the diurnal oscillations. While Hofrath Schwabe was engaged in delineating the sun-spots, Sir Edward Sabine was conducting a series of observations with regard to these spasmodic affections of the needle, and he found that such fluctuations are most frequent in years of high sun-spot activity." Nearly a hnndred years ago, Van Swinden had suggested a periodicity in these irregular magnetic movements. Gauss, Arago, Lamont, and Gautier, pursued the research, and established the existence of a cycle of magnetic variation having an eleven-year period, the maxima and minima agreeing with the maxima and minima of sun-spot activity. Schiaparelli and Broun have confirmed these results, and the latter observer concludes that, while the sun-spot activity is not an exact measure of magnetic action, "each is a distinct result due to the same cause." This disturbance is so great that, in years of maximum sun-spots, the working of the telegraph has been powerfully interfered with.—Prof. Youmans, *in Popular Science Monthly for January.*

### THE FORCE OF LANGUAGE.

A recent historian of Rome, toward the close of his famous attempt to undeceive the world at large with respect to the genius of Cicero, sums up his argument in the following words: "Ciceronianism is a problem which, in fact, cannot be properly solved, but can only be resolved into that greater mystery of human nature—language, and the effect of language on the mind."

These words are suggestive—suggestive, too, of a wider question that at first sight appears. That men are influenced by language at least as much as by ideas; that power of expression is intimately associated with mental grasp generally; even that a fascination is exercised by style to which nothing equivalent is found in the accompanying thought—these are acknowledged truths, readily granted. But it is a most singular thing that they are so readily granted; it is singular that the question is not oftener asked, "Why is this so?"

How is it that language, which is but the vehicle of thought, comes to have a force which is not the mere weight of that which it carries? Even where this is not the case, where there is an equivalence of value in both style and ideas, great conceptions being nobly expressed, how is it that the matter and the form seem to have independent claims upon the attention? In a word, what is that in language which is not mere *expressiveness* of the obvious intentions of the writer, but is yet a merit?—T. H. Wright, *in Popular Science Monthly for January.*

Is it not due to the poetical element in our uature, that instinctively enjoys the sound not less than the meaning of words?

### TELEPHONIC AUSCULATION.

A writer in the Medical Press and Circular says: "Last night I listened to a young lady's chest with a telephone; the young lady stood in the hall, and I was in the dining-room, thirty feet away. One cylinder was placed on the chest and the other at my ear, the connecting thread of the little toy being kept quite tense. I heard the healthy sounds of a very healthy chest quite distinctly."—*Louisville Med. News.*

